# Patients' Views With Regard to Personal Recovery in Forensic Psychiatry in German-Speaking Switzerland—An Explorative Study

**DOI:** 10.3389/fpsyt.2021.695096

**Published:** 2021-07-01

**Authors:** Susanne Schoppmann, Joachim Balensiefen, Steffen Lau, Marc Graf, Henning Hachtel

**Affiliations:** ^1^Departement Education, Research and Practice Development, Universitäre Psychiatrische Kliniken Basel, Basel, Switzerland; ^2^Department of Forensic Psychiatry, University Hospital of Psychiatry Zurich, Zurich, Switzerland; ^3^Forensic Department, Faculty of Medicine, Universitäre Psychiatrische Kliniken Basel, University of Basel, Basel, Switzerland

**Keywords:** personal recovery, forensic inpatient psychiatry, focus groups, attitudes, procedural justice, heteronomy, self-determination

## Abstract

Recovery orientation (RO) is a modality of supporting patients to improve self-determination, leading a meaningful life and well-being in general. This approach is widely studied in general psychiatry, but evidence is lacking for forensic inpatient settings in Switzerland. While secure forensic clinics tend to be regarded as total institutions, which are an anathema to RO, a project to implement RO interventions in this setting was financed by the Swiss Federal Office of Justice. This explorative study investigates baseline expectations and views of patients in forensic wards in German-speaking Switzerland in the context of a recovery-oriented intervention. As such wards are non-existent in Latin-speaking Switzerland, the investigation could only be carried out in this language region. Six focus groups with 37 forensic inpatients were conducted. Thematic analysis revealed two major and several subthemes. The major theme “heteronomy” includes the subthemes “stigmatization and shame,” “coercion,” “lack of support,” “mistrust,” “waiting,” and “structural impediments.” The subthemes “learning to live with the disorder and working on oneself,” “participation,” “connectedness,” “confidence,” and “joie de vivre” belong to the major theme “regaining self-determination.” In this way, results of prior research are extended to forensic peculiarities. Furthermore, the personal views of patients are discussed in detail regarding their possible influence on therapeutic outcomes and personal recovery. These findings should be of help to therapeutic staff in the respective setting to be better informed about, and to counter the effects of, heteronomy and long-term hospitalization. Important in this regard is the concept of procedural justice and the subjective client's perception thereof.

## Introduction

Putting a focus on personal recovery is well established (1) in international psychiatric care systems. Personal recovery is described as an individual process (2), which enables persons affected by mental disorders to make experiences that support and promote the aim of living a meaningful and self-determined life. To that end, the experience of being connected with others, having hope and optimism for the future, gaining or rebuilding a positive sense of identity, being empowered to make own decisions, and discovering life as meaningful are conceptualized as the most central elements. Although recovery orientation (RO) is widespread in Switzerland (3), e.g., in terms of established peer support, secure recovery (4)—i.e., using recovery principles in forensic mental health—is presently not systematically introduced, in contrast to other countries (5–10). In Switzerland, there are still doubts whether the promotion of empowerment and the challenges of security and control are compatible (5) under the given legal and therapeutic conditions.

Internationally, the body of empirical knowledge concerning secure recovery increases. Narrative synthesis of qualitative studies (9, 10) show that connectedness with family, friends, and supportive staff as well as the process of coming to terms with one's past and finding a sense of one's self in a safe and secure environment are important factors in supporting personal recovery. Drennan and Wooldridge (8) developed a briefing about making recovery a reality in forensic settings and identified five key areas, which could be of help in developing a recovery friendly climate. These key areas are supporting recovery along the care pathway, the quality of relationships, risk and safety, opportunities for building a “life beyond illness”—meaningful occupation, and peer support. Their comments on the key areas reflect the important factors mentioned above and show ways of putting them into practice.

Furthermore, and from a theoretical point of view, it can be suggested that a successful recovery orientation supports the long-term legal probation of patients. For example, there is well-documented experience with the Good Lives Model, which is applied in the treatment of sex offenders (13) and pursues similar goals as RO (14). There is also conceptual overlap with the concept of desistance, understood as refraining from substance use and/or criminal behavior, which has been used successfully in the treatment of persons with addictions and histories of offensive behavior (15, 16). Moreover, Martin and Stermac (17) found a correlative negative relationship between the risk of recidivism into criminal behavior and the presence of hope in a study of one hundred incarcerated men and women in Canada. Thereby, “hope”—as the realization that “things might get better”—is central to the idea of RO.

This paper reports the results of the analyses of six forensic inpatient focus groups, which have been held between February and June 2020 in two corresponding University hospitals for forensic psychiatry in German-speaking Switzerland. These focus groups are part of a mixed method project, which is—with a share of 80%—funded by the Swiss Federal Office of Justice, and aims to gain knowledge concerning the applicability of RO attitudes and interventions in forensic wards and the effectiveness of these adjustments on several factors of therapeutic relevance. This knowledge, in turn, should result in recommendations for the implementation of RO in forensic mental health institutions in Switzerland. In the context of the whole project, the results of the six focus groups in the following represent the qualitative part of the pre-intervention data survey.

## Materials and Methods

The focus groups are part of a larger (and still ongoing) project, which uses a pre–post mixed method approach (18, 19) in order to assess the effectiveness of RO interventions in the corresponding six medium secure wards. Before the focus groups were held, the two research fellows (SS and JB) had already had contact with the patients and the staff during a joint RO training session in each participating ward. Thereby, the research fellows introduced RO as a working principle in a psychiatric context and gave a brief outlook on the further proceedings of the project. The session focused on the theoretical principles of RO, which have been illustrated by a case report.

At the end of the session, participants of the joint training were asked if they were willing to take part in the qualitative and quantitative data analysis via focus groups and questionnaires. All potential participants received oral and written information about the project. Inclusion criteria were the ability to read, speak, and understand German sufficiently and the provision of written informed consent. The only exclusion criterion for patients was an acute mental health crisis. Slightly more than half of the patients of the joint training participated in the focus groups, whereas approximately two thirds of the total number of patients in all wards were present in this training. The patients received a small allowance for expense for participating in the data collection.

The Ethics Committee Northwest and Central Switzerland confirmed that the research fulfills the general ethical and scientific standards for research with humans.

### Theoretical Background

The (pre-)intervention focus groups are part of a mixed-method approach. Mixed-method research has its root in pragmatism (18, 19) and allows combining different methods of data collection and analysis in order to explore complex phenomena with not only the strengths and shortcomings of a single-method approach. Apart from the focus group data, the project includes (as a second type of qualitative data) minutes from the ongoing moderated workgroup sessions and the participatory observations. Finally, the project will include not only pre–post focus-group data but also quantitative pre–post questionnaire data after the post-surveys.

The documentary method (20, 21)—a method that is assigned to reconstructive social research—was seen to be appropriate as a theoretical base for the focus groups. As a premise, documentary method states that focus groups share a conjunctive knowledge about existing commonalities. In a focus group situation, the conjunctive knowledge—which is specific to a certain milieu—can be revealed. Thereby, the process of articulating conjunctive knowledge is self-directed, and no leading external interviewer disturbs the group members' access to their common experience by a thematically structured approach (20). Furthermore, and in contrast to individual interviews, the documentary method allows the distinction between idiosyncratic narratives of single interviewees and the dimension of conjunctively shared knowledge as the central subject of interest. Originally, the documentary method is directed to deducing theories from the material gained from the inductive process of generalizing patterns or types (21). Since our research, however, is directed on exploring experiences and expectations of forensic patients with regard to RO (and not on deducing a specific theory), we decided to use thematic analysis (22, 23), which is also applicable to data generated in focus groups (24).

### Participants

Six focus groups with a total of 37 patients were being conducted; thereby, most of the patients (*n* = 30) were male, which roughly reflects the common sex ratio in forensic wards. Participants were recruited from two University hospitals (Psychiatrische Universitätsklinik Zürich and Universitäre Psychiatrische Kliniken Basel), whereas each hospital was represented with three medium secure forensic wards. Between four and seven patients participated in each focus group [i.e., the recommended number of participants (25)]. One of the wards in Basel was a ward for adolescent patients.

The treating psychiatrists made the general routine diagnoses reported below according to ICD-10. These diagnoses largely reflect the diagnoses that have already been found in forensic institutions in Switzerland (11, 12).

The median age of the adult patients was 33.50 years (IQR = 12.00), ranging from 21 to 60 years. Most of the adult patients suffered from schizophrenia, schizotypal, and delusional disorders (89.9%), followed by mental/behavioral disorders related to the use of psychoactive substances (4.1 %), personality disorders (2.0%), behavioral/emotional disorders with onset in childhood and adolescence (2.0%), and affective disorders (2.0%). The median length of stay on the ward was 23.00 months (IQR = 32.75) and ranged from 1 month up to 15 years. Adult patients have been sentenced for violent offenses (69.4%), sexual offenses (12.2%), property offenses (10.2%), and other offenses (8.2%).

Regarding the adolescent patients (*n* = 4), the median age was 17.50 years (IQR = 3.00), ranging from 16 to 19 years. The adolescent patients suffered from personality disorders (50.0%), schizophrenia, schizotypal, and delusional disorders (25.0%) and behavioral/emotional disorders with onset in childhood and adolescence (25.0%). The median length of stay on the ward was 12.75 months (IQR = 8.25) and ranged from 3.5 up to 14.00 months. Adolescent patients were in the wards due to violent offenses (50.0%) and sexual offenses (50.0%).

## Procedures

### Proceeding in the Focus Groups

The interviews were held in conference rooms, located in premises of the forensic wards. Two research fellows, one as an interviewer (SS) and one as an assistant and observer (JB) (25), led the focus groups. The participants knew the researchers only from the joint training and had no other relationship to them. Staff members of the wards were absent during the focus groups, and anonymity concerning the participants' statements was guaranteed. Before the start, the participants gave written informed consent. The focus groups took between 60 and 75 min and were audiotape recorded. The participants were informed that the focus group does not have the purpose of answering specific questions, but rather to get in a thematic discussion with each other. As a narrative-generating prompt, the joint training was followed-up by the statement: “You all participated in the joint training, which introduced the concept of recovery-orientation. What about the training remains particularly memorable to you? What do you associate with it?” The interviewer occasionally asked questions of clarification or summarized the participants' statements. However, since the patients quickly came into self-directed discussions, only few interventions were necessary.

After the focus groups, the patients received a small allowance for their participation and were told that they would receive the results of the analyses in a few weeks. The research fellows (SS and JB) shared their impressions and made field notes and sketches of the seating arrangements immediately after the focus groups.

### Data Analysis

The interviews of all focus groups were transcribed verbatim. The analyses of the focus groups were based on thematic analysis (22) in an inductive way without using a pre-existing coding frame. The transcripts were read several times and compared with the audio files, in order to control for transcription errors and to become familiar with the material. Afterward, all passages were coded, and the codes were collated in order to find global themes specific to the individual transcripts. Last, the whole data set—i.e., all the individual transcripts—was analyzed and reviewed for thematic similarities and differences. In the course of this last step, themes were repeatedly adapted or changed in discussion with the research team (SSC, JB, HH).

### Trustworthiness

At least two (SSC and JB) researchers analyzed all transcripts independently. At all stages of the interpretative process, the primary investigator (SSC) reflected on her own experience using reflective writing (26). The results were taken back to the wards and presented to patients and employees. The patients confirmed that the results reflected their own impressions concerning the interviews' content and expressed gratitude that their voice was heard. In preparation of the paper, the COREQ-criteria (27) were being used. All interview quotations in the following were translated in an attempt to reflect the patients' original wording as close as possible.

## Results

In all focus groups, the participants compared memories of the joint training session with their current situation, which was perceived as a situation of external determination and personal resignation. During the focus groups, the participants began to negotiate the possibilities of (re)gaining self-determination over their life. Against that background, the thematic analysis revealed a bipolar structure, whereas its two poles were defined by the vertical global themes “Heteronomy” and “Regaining self-determination.” Each of those global themes contained several subthemes, which were horizontally interlinked in a specific way: The subthemes of the latter reflected possible ideas and solutions to problems that have been elaborated in the context of the restrictions in a medium secure forensic ward.

Table 1 shows an overview of themes and subthemes.

**Table 1:** Overview themes and subthemes.

**Heteronomy**	**Regaining self-determination**
Stigmatization and shame	Learning to live with the disorder and working on oneself
Coercion	Participation
Lack of support	Connectedness
Mistrust	Confidence
Waiting	Personal development and joie de vivre
Structural impediments	

These themes and subthemes shall be presented and commented by citing quotations, which represent the content of the other quotations that have been subsumed under a specific subtheme throughout the thematic analysis.

Before presenting the themes, it should be noted that the participants vividly remembered the joint training session, since it provided hope and motivation for a change. Exemplary, this instillation of hope and optimism is portrayed in the following piece of dialogue between two participants:

“*P1: A motivational injection. Yes, it has given one hope*.

*P2: Yes and strengthened the will to see for myself what I can do for my future, that has already given a strength of will.”* (FG 2, L. 45–49)

Furthermore, the participants emphasized the value of the fact that the joint training was directed at both patients and staff members as equal partners in a scientific project, since this experience was perceived as a break with the conventions of the monotonous everyday life procedures of a forensic ward.

### The Current Situation: Heteronomy

The following subthemes are subsumed under the umbrella term “heteronomy”: stigmatization, shame, coercion, lack of support, mistrust, waiting, and structural impediments.

#### Stigmatization

The analyses revealed that different types of stigmatization were experienced. At the beginning of the focus groups, self-stigmatization became indirectly evident in the fact that some participants expressed astonishment that the case report that had been presented in the joint training session had succeeded in leading a crime-free, self-determined, and fulfilling life after many years as an inpatient in forensic psychiatry, something that some participants hardly dared to imagine for themselves:

“*He certainly is a prime example. He has been able, hasn't he? He has had the strength to pick himself up and learn a trade, right? I would—I am sure not everybody can do that, right?”* (FG 1, L. 35–37)

Other participants apologized for being a not competent participant due to a lack of general knowledge. At the end of the focus groups, participants often asked whether they had done everything correctly or if they might have complained too much. This uncertainty reflects a lack of self-confidence, probably due to self-stigmatization. Moreover, the participants reported experiences of structural stigmatization:

“*P1: Yes, it's clear, of course. If something happens again, everyone is afraid. And afterwards—it is true: they throw us into the same boat. Afterwards we have a collective punishment*.

*P2: And that is pretty shitty. Because you're under the penal code—You're doing everything right. I—Yeah, you're doing it right with the outlets and so on*.

*P3: And that just doesn't work in Switzerland. In Switzerland everyone marches to a different beat, so to speak, the higher ones, who are in the head to head in it—The authorities think—Yes.“* (FG 3, L. 215–223)

This quote shows that the double stigma—mentally ill and dangerous—will, according to the participants' perception, always stick to them, even if they make no mistakes.

Sometimes, participants partially felt stigmatized by employees in terms of accusations when a deterioration of their ill health had occurred. Instead of feeling understood in their state of illness and accompanied in a hope-inducing way, they felt just dispatched with medication, and urged to justify their condition:

“*Yes, you're almost depicted as the guilty one if you are having symptoms of illness. You're just expected to function. And if something happens, you're almost grilled, just “You're not doing so well again,” and instead of showing accompaniment and support somehow, yeah, that has to be right now. It's just, you're just expected to function like a normal person, and if that doesn't work, administer more medication. Honestly, that's the image I have of this hospital here.”* (FG 2, L. 391–402)

Against that background, the general impression is that they have little chance of actual resocialization:

“*Exactly, somewhat startled I have noticed that in many cases it is like that, and you only tell about life up to that point* [meaning the offense committed]. *And yes, the rest, you can't write that in a resume: I was now also in forensic psychiatry.”* (FG 2, L. 23–27)

#### Shame

For one thing, the last quotation outlines the consequences of structural stigmatization; however, it also points to a moment of shame. It was striking that the offenses of the participants were merely mentioned and remain “behind a veil” even for themselves, as one participant put it. After the interviews, other participants expressed concern that they would be urged to talk about the offense they had committed, which was not the case. Lifting this veil requires a great deal of sensitivity from the mental health workers and an appropriate pace in dealing with the offense in order not to risk an inner or actual breakup of therapy by the patient. The most specific statement about an offense was the following:

“*I did a crime without intending to actually hurt anyone, right? But I still hurt someone, right? And I'm going to get locked up.“* (FG 1, L. 39–41)

The statement served as an introduction for the participant to share his thoughts on compulsory treatment.

#### Coercion

The participants discussed coercion and pressure, as well as bad experiences with psychiatry, in general, in many ways. A distinction must be drawn between coercive therapies, coercive measures (such as forced medication and isolation), and informal coercion, (which participants refer to as “pressure”). The feeling that coercion is applied in a disproportionate way was expressed in several focus groups and refers not only to individual measures but also to the system as a whole. Especially the duration of the detention was perceived as being disproportionate and therefore unjust.

Being treated in a forensic psychiatric hospital for therapy is an ambivalent issue: On the one hand, the realization of being treated in forensic psychiatry has a devastating effect; on the other hand, there is hope that therapy might empower one to make something of one's life. The following quote shows the whole ambivalence:

“*Yes. You can't get much further down than that. And then you get things like: I don't want this. And rebelling against forensics and against what's being done here. And on the other hand, you want to go to therapy. Maybe with reluctance, because you have the feeling: actually, I don't want to do this at all. And I am simply forced into a therapy that the team sees as good for the patient. But I don't feel that way myself.”* (FG 3, L. 50–55)

Furthermore, some participants perceived being locked up for a crime as being acceptable, but not being forcibly medicated against one's will. In some instances, this was considered as an illegitimate intervention concerning a patient's body:

“*[...] And I am locked up. And the worst is forced medication or against my will. I don't know how to assess that. I'm not a doctor. The effect of the medication on me, right? And, yeah. That's a tough one, isn't it? I think that forced medication violates basic rights. Because the body belongs to me. It doesn't belong to the state. It can't dispose of me, can it? It can lock me up for a crime. But it cannot dispose of my body. So, yes. That's difficult.”* (FG 1, L. 41–46)

Being confined restricts movement, but forced medication invades the body, and (that, at least, is the participant's fear) possibly changes the personality.

In addition to formal forms of coercion, there is pressure, which can refer to the process of achieving therapeutic goals. If these goals are not achieved, some of the participants felt to be put under pressure:

“*Yes, the expectations are to achieve goals. And if it doesn't work, they don't ask what the problem is. Instead, it's said, “You have to” instead of communicating with each other about this issue. It is always—how shall I put it? It's defined what we have to do and not talked about what makes it troublesome to achieve it. If goals are not met, there is no support, there is more pressure.”* (FG 2, L. 350–355)

Thereby, the participants emphasized that they would like to be adequately challenged instead of being overburdened (in this context, it might be critical to note that the focus of the corresponding statements was not on questioning the need for insistent requests in certain situations, in general).

#### Lack of Support

Lack of support was described in relation to therapy. Some participants experienced therapy as very standardized and not individually adjusted enough to provide any real help; thereby, especially psychotherapy was experienced as being too superficial and as being carried out not often enough. However, other patients did also emphasize that the staff focused on the resources and strengths of the patients and made efforts to strengthen them.

Another perceived lack of support related directly to recovery orientation. When it comes to finding new ways, one needs the support of people that point out paths and accompany them for a while.

“*Yeah, I don't know, with recovery it's kind of finding a new way, isn't it? And that's already… That can be a problem, when you're in such a dead end, you have no motivation and nothing, you can't think of anything, then you really need someone who can show you ways somehow. And here, most of the time it's just like, “Oh, you're doing not well, yeah, medication.” Instead of being shown a way, just something.”* (FG 2, L. 187–191)

Particularly against the background of a criminal biography, knowledge about developmental necessities or educational opportunities are often not available. In this respect, the participants depend on the support of the staff members in order to face achievable goals and possibilities. From the participants' perspective, this does not happen often enough. Furthermore, the focus groups revealed that own ideas—such as working as a freelancer again—are sometimes considered to be unrealistic from the outset or even interpreted as a worsening of symptoms of illness. The perceived lack of support may also lead to mistrust toward the staff members.

#### Mistrust

On the one hand, mistrust applies to psychiatry, in general, and is closely linked to the history of psychiatry and what the participants know about it:

“*So, for example, one point is that psychiatrists are working with pharmaceutical companies. Right? And last year—I think it was last year—they brought on the news, in various hospitals they have done studies on patients for decades, without their knowledge. Did you notice that?”* (FG 1, L. 358–361)

Consequently, the participants wish for an ethical authority that controls forensic psychiatrists and forensic psychiatric hospitals. This demonstrates that there is a perceived need for protection from arbitrariness.

The impression of being exposed to the arbitrariness of the authorities and the staff members' results from the relationship of dependency concerning the forensic–psychiatric system and the resulting experiences. Thus, several participants reported that they do not openly communicate their feelings (e.g., regarding their state of health) since some of them have experienced that medication may be increased and freedom of movement restricted as a consequence:

“*Above all, he* [meaning the doctor] *writes as he thinks. It's cursed. You don't know what to say. If I say I have suicidal thoughts, then maybe it means off to the isolation room.”* (FG 2, L. 246–247)

Mistrust leads to less openness toward the staff members, which—in turn—might cause the provision of delayed or lacking help and support. This association became particularly clear regarding the handling of suicidal thoughts: keeping them secret due to fear concerning physical isolation might lead to psychological and emotional isolation, since the patients remain alone with their condition.

Furthermore, the imbalance of power between staff members and patients was addressed many times.

“*Patients are in a position where they are dependent on those who have the key. And you must have a good feeling about that. That the one does not want to harm you.”* (FG 3, L. 531–532)

Although the participants said that they became familiar with the staff as time continued, it would remain difficult to trust them because they not only have the key but also judge the risk of relapse, which has a direct influence on the length of stay.

#### Waiting

The subtheme of waiting was a very dominant one and contained several aspects. Most importantly, “waiting” often referred to the end of the measure and the discharge from forensic psychiatry. However, the patients in forensic psychiatry usually do not know in advance when they will be released. The participants perceived that as unfair in comparison with prisoners in jail.

“*Because someone who is in prison also knows how long he must serve, right? He can put the daily lines on the wall and every day is one less. I have no idea how long I'm sitting here, I'm in uncertainty, because I'm always just waiting, waiting, waiting until there maybe once a therapy talk, and then I come a tiny step further in a treatment plan conference, where the authority is not even there.”* (FG 3, L. 655–659)

Moreover (and consequently), the pursuit of life goals is postponed until after the release because the patients see little opportunities for personal growth during treatment. How long one must wait depends—among other things—on whether the behavior meets the expectations of the staff. If it does, stepwise easing is achieved more quickly:

“*But, for example, if you bully someone, and they tell the nursing staff, then it doesn't go as fast with the easing, for example, then you have to wait longer and stuff. So, it depends on how you behave on the ward. That plays quite a role, yes.”* (FG 2, L. 618–621)

Cooperation between forensic psychiatric institutions and judicial authorities also seem to have a major impact on the waiting time. Some participants stated that it took months (and in some cases, even years) before the corresponding responsible persons of the psychiatric and legal system met in order to talk about their cases.

Waiting is closely related to monotony and boredom. The daily and weekly rhythms are repetitive, and even the guided walks take place on the same paths, which often cause the impression that time stands still.

“*Or that it's so monotonous on the ward, the same thing every day. Always the same thing. Sometimes you think to yourself, “What should I do?” But you just don't get anywhere. It's the same every day, every day the same routine from morning to night, from Monday to Sunday always the same. [...] We sit here and time seems to stand still. The only thing that is on is the television.”* (FG 2, L. 519–555)

This monotony can lead to lethargy. As an exception, patients in the ward for adolescents rather spoke about boredom than monotony, but described the feeling in a similar way:

“*Yes, no, but you.I also have the feeling outside, if things are repeated over and over again, then it's boring, now everywhere there's news again, the things with the Corona virus, with time you just can't hear it anymore. And on the ward it's mostly the same. When things happen, then it could, yes, turn people against each other. But it would be exciting, then something is going on.”* (FG 5, L. 114–119)

It seems as if adolescents do not surrender to boredom, but counteract the feeling through the evocation of conflicts.

#### Structural Impediments

Structural difficulties, such as features of the spatial constriction, lack of opportunities for retreat, and a lack of privacy, were discussed in only one hospital. In this hospital, patients are predominantly in two- or three-bed rooms, whereas the only opportunity for privacy is a timeout in one of the isolation rooms. The following quote reflects much of what was said in the interviews in one sentence:

“*This is horror, you can never be alone.”* (FG 4, L. 513)

Living together in a confined space requires a lot of mutual consideration, which is not always given in the forensic wards. Therefore, the spatial confinement is perceived as an additional stress factor. Consequently, many patients have the impression that it is hard not to get sicker than they already are. This is hard work: It requires distancing oneself from the moods and conditions of fellow patients.

“*And so you're just always in the state of change. Right? Because, the team changes. You also have to reorganize yourself day by day. You do not have the peace and quiet that you actually need. Because, for example, in the isolation rooms, when two are occupied, the doorbell rings. Then someone is shouting. Another one is angry. Not to let all that get to you, that you don't become sicker than you are, is a hard task.”* (FG 3, L. 61–66)

Failing to do this job might mean an increase in medication, which, however, has no effect on the social stress level and the lack of possibilities for retreat caused by the institution.

### Regaining Self-Determination

So far, the experience of heteronomy has been described as the dominant situation. Although the discussion of particularly this situation took considerable time, the participants also repeatedly expressed hope for a change by contributing thoughts and ideas on possible ways to regain self-determination. However, this often seemed to be beyond the realm of possibility, as the following quote shows:

“*I just thought of that one, too*. [Meaning the case study used in the training] *And when I saw that tears came to my eyes. There at that session. Because I felt like, wow, there really is still a real chance to get out of a system like forensics.”* (FG 3, L. 42–45)

In this respect, the training provided hope, which reflects the belief that it is possible to lead a life outside of forensics. The participating patients perceived this as a motivation to pursue this goal.

In the following, the subcategories of the theme “regaining self-determination” are presented: learning to live with the disease and working on oneself, participation, connectedness, confidence, personal development, and joie de vivre.

#### Learning to Live With the Disease and Working on Oneself

“Learning to live with the disease” was a dominant topic in all focus groups. Based on the case report in the training, participants concluded that their recovery will not be a matter of medical recovery, but a matter of learning how to live a meaningful, self-determined, and satisfying life with the disease:

“*Yes, you do not get in healthy. But you don't come out healthy either. You simply have to deal with the fact that the disease is there. And that you know how to live with this disease.”* (FG 3, L. 346–348)

Learning to live with the disease includes knowledge about its manifestations and symptoms, which is usually provided in psychoeducation. However, if this knowledge is not available, it might happen that a state of health worsens without subjective notification. Medication was generally considered to help living and remaining stable, despite all controversial discussions concerning the application of medication and skepticism about the side effects in the focus groups:

“*Yes, I'm dealing with that better. With the anger, and with the violent fantasies, and with the delusions. With hearing voices and seeing images. I haven't had that for a few months now because I'm well-adjusted with the medication and because I recognize my early warning signs correctly.”* (FG 1, L. 144–148)

The participants appreciated that the treating psychiatrists usually pay attention to a good medication regime. However, regaining self-determination involves more than that. It also involves working on oneself concerning the reappraisal of one's own history and—in the process—facing up to work on delinquency, a task, which is often considered to be very hard:

“*Then I found a hiding place, where I then locked myself away again. And here is where I'm now working through my offense. Where I really have a good pace. Where I don't have this hiding place. And I still think that's good. […]”* (FG 4, L. 118–122)

This task might require time which is “sufficiently available” in forensic psychiatry. Thus, some participants—despite the desire for self-determination—understand that the process of spending a long time in forensics is a presupposition for the opportunity to become aware of the condition concerning ones future against the background of mental health problems and a history of offensive behavior.

#### Participation

Primarily, participation meant being involved in decisions. This involved, for instance, ward rules or the consumption of medication. However, participation was also related to the act of acknowledging the patients' expertise for themselves and giving them the confidence to try things out:

“*Yeah. So, just not more demands. Now I have to do more skills and withdraw more and so. That I'm trusted a little bit more, even if I have symptoms sometimes. That it's like that for a moment. And when I notice that it's getting too much for me, then I simply withdraw automatically. That I'm trusted to do that.”* (FG 1, L.531–536)

There appear to be situations in which the participants feel overwhelmed by the staff members, e.g., when they are expected to do something that they cannot do due to symptoms of illness. In these situations, they wished that their own expertise would be recognized and that they would be supported regarding the question on how to fulfill the requirements:

“*P2: Yes, for example [...] in occupational therapy, where I am in occupational therapy, he tells me, “You always work unfocused.“, and so, he expects me to be focused. And I've told him before, quite frankly,” listen, I live with residual symptoms. It's not easy for me to work with these residual symptoms.” That's one example, yes*.

*P3: Instead of helping him how to achieve the goal and just stay focused*.

*P2: So he expects more effort from me in that sense, but it's not like I'm not working.”* (FG 2, L.358–369)

As this sequence shows, participants would like to be promoted but not overwhelmed.

Moreover, some participants claimed that they would like to participate more actively in the treatment-plan conferences or the site assessments (in some wards, patients participate in these procedures; in others, only partially, and in some wards, not at all). However, there was a clear wish for presence in these procedures from the beginning:

“*P1: No, but I think it's important to be there from the beginning, then you're not so surprised about what you're going to do*.

*P2: Already be surprised at the treatment plan conference, but at least it is agreed before you, and not so...”* (FG 5, L. 230–232)

Furthermore, some participants demanded the presence of representatives of the legal authorities in these proceedings. In some cases, the participants are not even personally acquainted with them, although they have a far-reaching influence on their lives. Last, though there was also an understanding that staff and authorities should be able to exchange information in the absence of patients, the desire to be present in these meetings concerning their person predominated.

#### Connectedness

The concept of connectedness represents the desire for connection with and to other people. The participants experienced their corresponding social situation predominantly in a mode of deficiency. Some participants indicated that they do not have any contact with family, former friends, or acquaintances any more. Particularly, availability of contact with family members was considered to be important and supportive.

“*I: But you all may still have social connectedness with other people who are not in the hospital, right?*

*P 1: I no longer. I've lost everything, haven't I*.

*P 2: Me too. I don't have anything anymore*.

*P 3: I have the family environment, right. But otherwise with the people I had to do with when I lived outside, I have no contact anymore*.

*[…]*

*P 4: So family is simply important. It's the best support you have. Because, family brings – friends and so –, yeah, not necessarily. Now and then. But family is the most important support.”* (FG 1 L. 224–250)

Maintaining ones' social relations is difficult under the conditions of forensic psychiatry. However, the participants saw a possibility to improve the contact to the outside world throughout the permission of a more extensive use of the available means of communication, such as telephone and Internet.

Regarding the state of being socially connected, the bond among each other in the group of patients also played a role. However, friendships within the patients appear to be rather frowned upon in some wards:

“*That's another thing, isn't it. You live with 12 different, with 13 different people in one house. Then it's clear, you know each other with the months and years, that you get to know each other a little bit. Then you can't say quite simply, no friendship or nothing. We are yes—we have a heart. If you look. We are, after all, people. Times binds something, right. So collegialities or something. You can't avoid that.”* (FG 1, L. 214–219)

Friendships and familiarity with each other cannot be prohibited. They arise due to the spatial proximity and temporal duration of the stay. This familiarity might also develop over time with the employees, which can easily seduce patients to think of them in terms of “colleagues.”

“*The question is always, at some point you look at them as colleagues. Yes, but that backfires.”* (FGI 2, L. 260–261)

Even when a certain familiarity with team members develops over time, this is no substitute for an equal trusting relationship, since the staff has the key for opening and closing doors both literally and figuratively. Nevertheless, a wish for more confidence to the employees was clearly present.

#### Confidence

Trust in the treatment and in the employees might be achieved through more institutional transparency, as patients were hoping. “Transparency” also includes openness on the part of the team members. This might lead to more trust and more positive feedback and praise from the team, which in turn would empower patients' self-confidence:

“*That would change something with the openness. That would perhaps also promote trust when you communicate with each other. It would be good because that is part of what is actually often missing [...]. Communication between patients and nurses is mostly really in case of crises or when something is going badly, but actually practically not if something is going well. I believe it helps people actually very firmly, if one gets not only negative feedback, but also positive, but most are just rather focused on the negative.”* (FG 5, L. 245–268)

Moreover, more positive feedback might result in an improved group cohesion with the staff on the ward as well. On the other hand, staff might have to devote more time to the patients, e.g., by playing games and talking, which could also contribute to the feeling of connectedness and confidence.

The desire for a trusted person was very present. This confidant could be a peer person who—as a peer—would not have to make predictions about dangerousness and risks of relapse, so that it might be easier for patients to be open. However, some patients claimed that this peer would also need to have firsthand experience in forensic psychiatry in order to understand how the patients are doing:

“*They're all different* [meaning peer workers in general psychiatry]. *It's not the same thing. To me, a peer worker means they have been through forensics themselves.”* (FG 3, L 154–155)

A peer worker with an own forensic background might better understand the stigma and shame associated with the offense and know what the monotony and waiting feels like.

For participants, the monotony might also be interrupted by activities associated with personal development and enjoyment of life, which would make the waiting more tolerable.

#### Personal Development and Joie De Vivre

Personal development and enjoyment of life were linked to activities that overrule the uniformity of the days and give meaning to the process of waiting. This included education and learning. Several participants reported that they miss the possibility of pursuing an education or further development in school during their inpatient stay:

“*Exactly, that's what I wanted to address. Here you have like no opportunities to move on, to do training, or any training, apprenticeship or anything. You come here and then you're just here, and then you come out and work in a supported workshop. Very few, on the other hand, can do anything else on the job*.” (FG 2, L. 443–447)

This was not necessarily about professional education, but about the possibility to educate oneself or to raise one's own level of learning. For instance, the joint training on recovery was being cited as an example:

“*Doing continuing education. Like staff training. That we can also continue our education and so. That there is further training. That take place on the ward, on the department. Like this information–that you did on Monday. Something like that. That there are maybe one or two times a month. With different personalities and so. That would still be fun.”* (FG 3, L. 375–379)

Being able to develop personally would counteract the perceived waste of time by waiting and point to a future perspective.

Similarly, participants demanded for activities that are associated with joie de vivre. It is important that one has the possibility to experience joy because this helps to overcome boredom and resignation and keeps one motivated:

“*There are extreme problems that have arisen, especially with me, but there are other people who have psychological problems, and if nothing brighter comes, not something comes where they somehow. Mr. (…) tries it all the time with music, tries to do something on the laptop, he gets a new program, he plays the guitar all the time and he's been here almost 4 years now. And at some point, maybe the will is no longer so strong where you just. you get out of it.”* (FG 6, L. 377–383)

Such activities can be of a sporting or social nature; they can be nature experiences associated with freedom or social experiences while cooking together. The decisive factor is that they are associated with joy and pleasure. For instance, there were reports of joint activities such as a visit to a circus, which, however, occur too rarely according to the participants:

“*Yes, too, but it's too rare. [...] or go to the circus. But not everyone can go, because you need a certain level and so on. And that's just a bit of a hassle.”* (FG 2, L. 528−530)

In this example, the focus is on the commonality of the enterprise that takes place outside the ward. However, there are also examples of joie de vivre throughout connectedness with nature.

“*I'm, I've noticed that it's good for me. Everything is flat with me when I'm cooped up. When I get out, spend an hour outside, for example Nordic walking or walking in the woods, I get more volume in my heart and my awareness becomes sharper. I notice that very clearly. It does me a lot of good.”* (FGI 1, L. 474–477)

Finally, of course, enjoyable food creates joie de vivre. In particular, the adolescents said that they did not like the food in the hospital and wish they could cook for themselves every day. Because—from their point of view—that would give them a little more control over their lives and:

“*good food, that really makes you happy*.” (FG 5, L. 67).

## Discussion

First and foremost, our results reflect the aspiration of forensic inpatients to regain self-determination, whereas most of the broached themes and problems reflect the ones that have already been elaborated with respect to RO and psychiatry in general (28–31). This finding highlights—from a theoretical point of view—that there is little reason to suggest that forensic inpatients differ fundamentally from inpatients in usual psychiatric wards concerning the opening to work on an RO-base in the corresponding institutions. Moreover, and from a more general point of view, both the attempt to (re)gain autonomy and the effects of the perception of heteronomy reflect fundamental principles proclaimed in self-determination theory (32–34), according to which the experience of autonomy, competence, and relatedness are basic human needs, whereas factors that counter these needs provoke reduced motivation and performance.

Furthermore, the results highlight insights that are more specific and thereby emphasize an under researched aspect in one final respect.

For one thing, the fact that forensic inpatients suffer from dual or triple stigmatization is a well-known phenomenon (7–9, 35). While there are antistigma campaigns initiated from institutions like the World Psychiatric Association (36)—which is directed on fighting the stigma of schizophrenia in general—there are little attempts to reduce the stigmatization of forensic patients (37). It has already been revealed that stigmatization of psychiatric patients is not only mediated throughout public opinion but also throughout mental health practitioners (39). In this context, focus groups with schizophrenic patients were being performed (38); the corresponding outcomes indicated that patients felt stigmatized in their relation to mental health practitioners in terms of receiving mostly standard treatment that is focused on medication, whereas their personal needs and problems remained largely unconsidered. Our results reflected exactly these issues in a forensic context.

Furthermore, our results suggest a close connection between the psychological phenomena of stigmatization and shame in forensic inpatients: In line with this study, Askola et al. (40) revealed that forensic inpatients relate their feeling of shame to the corresponding offense. Shame can be defined as a sense of mental fear or pain in a moment of weakness, failure, disregard, devaluation, or scorn while being exposed to the perception of others (41). Regarding the characteristics of a pejorative social reaction, the feeling of shame thereby shares some basic properties with the phenomenon of stigmatization, since the latter consists in the perception (or internalization) of a pejorative perspective on a person. Moreover, in drawing a line between the feelings of shame and guilt, Blankenburg (42) points out that shame—in contrast to guilt—does not necessarily presuppose the impression of being responsible for the corresponding shameful action. Thus, it can be hypothesized that forensic inpatients, who are being treated as “insane offenders,” tend to process their offense by considering themselves as non-accountable, which evokes the feeling of shame (and not guilt), but thereby inevitably merges their identity with the stigma of a mentally ill person (self-stigmatization). Whereas, an offender feeling guilty by one's actions might have the prospect and confidence of changing one's behavior, an ashamed offender might feel his character or mental appearance as the primary fault, which would not be receptive for change. As a result, shame in therapeutic settings is a non-favorable emotional state. This asks for further analyses regarding the psychological mechanisms of shame, guilt, and stigmatization in forensic inpatients.

Second, the compulsory character of treatment in a forensic psychiatry is often perceived as coercion (31, 43), for obvious reasons. Instances of coercion are conceptually sub classified into instances of direct coercion (e.g., forced isolation or forced medication) and instances of indirect coercion (e.g., ward rules and corresponding sanctions). The extent of applying instances of indirect coercion, thereby, determines the degree of the perceived restrictiveness. Tomlin, Bartlett, and Völlm (44) conducted a concept analysis of restrictiveness in forensic psychiatric care systems based on 50 empirical articles and identified two fundamental factors. One of these factors is the inherent aim of the forensic system, which is either rather caring or patronizing. The other one, as an indicator for a restrictive system, is the dominant presence of the notion of risk management. Both of these factors have implications on an individual, institutional, and systemic level. As an example for a restrictive climate in individual respects, staff members are being described by patients as being “*key-holders, lacking in empathy, insensitive, disempowering, forceful, abusive, prone to over-reaction”* ((45), p. 34). The patients in our focus groups partly reflect these charges against staff members in terms of a lack of support and mistrust, which contributes to the perception of an indirectly coercive institutional level in the corresponding wards. Procedural justice might be a key to overcome the perception of coercion (31, 46), since the six crucial components of procedural justice described by Wittouck and VanderBecken (47)—namely, fairness, voice, validation, respect, motivation, and trust and information—partly mirror demands and request of the patients in our interviews.

Third, the process of waiting in its significance for forensic inpatients has been largely ignored by the literature up to date, though boredom—as a closely related psychological phenomenon—has received some attention (11, 12, 45), particularly in the context of occupational therapy (50, 51). Nevertheless, it is critical to emphasize that there is a conceptual difference between the process of waiting and the phenomenon of boredom. Whereas “waiting” refers to the anticipation of an event—in case of forensic inpatients, mostly the anticipation of the result of an application concerning a legal easing throughout a bureaucratic process, which they often perceive as being opaque—“boredom” can be defined as a lack of meaning (52) due to a lack of external stimuli (53). Consequently, boredom can result from the process of waiting for certain stimuli but is not synonymous to the latter. From a more general point of view, the psychological process of waiting appears to be a rather underexposed topic. Witowska et al. (48) report that the effects of waiting have been taken into scientific consideration exclusively in short-term waiting scenarios. The results of an associated empirical study suggest that boredom results from a lack of emotional and cognitive self-regulation in waiting situations, while “*time flies when you are having fun”* (48, p. 5). Furthermore, self-regulation is a crucial element of self-determination theory (49), whereas different modes of self-regulation are conceptualized as being critical for factors concerning behavior, social attitudes, and mental health. Considering the context information given above in the light of our thematic analysis suggests that the patients' wish for distraction regarding the monotonous everyday life in a forensic ward (articulated and discussed in the thematic dimension “personal development and joie de vivre”) reflects a request for opportunities of cognitive and emotional self-regulation. This request might be a psychological mechanism order to counter boredom that results (*inter alia*) from the process of waiting. Moreover, boredom is associated with feelings of anger and frustration (54) and might result in detrimental behavior (45) like absconding (55, 56), which then—in turn—might retard the process of personal recovery.

Finally, our results partially confirm the results of prior qualitative studies. In a narrative synthesis of five articles, Clark et al. (9) concluded that the themes “safety and security,” “the dynamics of hope and social networks,” and “work on identity as a changing feature” are the most central ones for initiating and supporting the recovery process. Shepherd et al. (10) identified six themes in a metasynthesis concerning 11 articles, namely, “connectedness,” “sense of self,” “coming to terms with the past,” “freedom,” “hope,” and “health and intervention,” whereas they considered the first two to be superordinate themes. Most notably, our analysis did also reveal “connectedness” as a central theme, whereas our themes, “stigmatization and shame” and “personal development and joie de vivre” reflect central problems and demands that can be assigned to the themes “work on identity as a changing feature,” “sense of self,” and “coming to terms with the past.” Moreover, Drennan and Wooldridge (8) describe the theme of personal development and joie de vivre, which is closely linked to the need for education and pleasant activities outside the ward, under the key area “building a life beyond illness—meaningful occupation” and conclude:

“*Meaningful occupation provides purpose, structure, routine and pleasure. These all contribute to a sense of personal agency (…) Filling time with personally meaningful activities restores a sense of value and purpose to life promoting hope and a belief that the individual can still pursue their dreams”* (8, P. 15).

This is what the participants in our study are longing for. However, all of the articles that were taken into consideration relate to wards in English-speaking countries. Thus, it might be that the themes “mistrust” and “waiting,” for instance, reflect specific characteristics of forensic wards in Switzerland.

## Strengths and Limitations

This explorative study assesses the experiences and expectations concerning RO in forensic inpatients in German-speaking Switzerland. Our results are consistent with thematically interrelated findings raised in an international context (8–10), which indicates the transferability of the findings.

Nevertheless, the results and implications of this study are restricted by some limitations. First, the focus groups were only performed in two forensic–psychiatric institutions in German-speaking Switzerland. Perhaps, interviews in other institutions might have yielded different results. Second, the participants participated on their own free will. Against that background, selection bias might have occurred. Third, neither expertise in qualitative research nor revealing and preventing techniques—like writing reflective diaries—can rule out the threat of subjective and intersubjective biases on the process of interpretation.

## Conclusion and Future Work

Applying principles of procedural justice to the therapeutic milieu and daily living of forensic patients is a promising candidate for supporting RO in forensic wards, as well as implementing recovery colleges (57), which might contribute to the gratification of the desire concerning personal development via education.

Furthermore, our results suggest effect relationships between the psychic mechanisms of shame, guilt, and stigmatization, and the psychomedical mechanisms of waiting, boredom, and negative health outcomes. Future research should investigate these relationships in order to ameliorate treatment conditions on forensic wards.

## Data Availability Statement

The raw data supporting the conclusions of this article will be made available by the authors, without undue reservation.

## Ethics Statement

The studies involving human participants were reviewed and approved by Ethics Committee Northwest and Central Switzerland. Written informed consent from the participants' legal guardian/next of kin was not required to participate in this study in accordance with the national legislation and the institutional requirements.

## Author Contributions

SS conceptualized the study, developed the methodology, was in charge of the investigation, wrote the original draft, and wrote, reviewed, and edited the manuscript. JB was in charge of the investigation, developed the methodology, and wrote, reviewed, and edited the manuscript. SL and MG conceptualized the study and wrote and reviewed the manuscript. HH conceptualized the study, developed the methodology, and wrote, reviewed, and edited the manuscript. All authors contributed to the article and approved the submitted version.

## Conflict of Interest

The authors declare that the research was conducted in the absence of any commercial or financial relationships that could be construed as a potential conflict of interest.
